# Treatment of Fabry Disease: Established and Emerging Therapies

**DOI:** 10.3390/ph16020320

**Published:** 2023-02-20

**Authors:** Muhammad Umer, Dinesh K. Kalra

**Affiliations:** Division of Cardiology, University of Louisville, Louisville, KY 40202, USA

**Keywords:** Fabry disease, left ventricular hypertrophy, enzyme replacement therapy, chaperone therapy, gene therapy

## Abstract

Fabry disease (FD) is a rare, X-linked inherited disorder of glycosphingolipid metabolism. It leads to the progressive accumulation of globotriaosylceramide within lysosomes due to a deficiency of α-galactosidase A enzyme. It involves multiple organs, predominantly the renal, cardiac, and cerebrovascular systems. Early diagnosis and treatment are critical to prevent progression to irreversible tissue damage and organ failure, and to halt life-threatening complications that can significantly reduce life expectancy. This review will focus on the established and emerging treatment options for FD.

## 1. Introduction

Fabry Disease (FD) is a monogenic X-chromosome-linked lysosomal storage disorder caused by mutations in the GLA gene, which codes for the lysosomal enzyme α-galactosidase A (α-GAL). These mutations result in an absence or deficiency of the enzymatic activity of α-GAL [1]. There are more than 1000 GLA gene variants, including pathogenic mutations, variants of unknown significance, and benign polymorphisms. The prevalence of FD varies from 1 in 40,000 to 1 in 117,000 (1) in the general population, depending on how rigorously screening is carried out to look for it. Moreover, FD is the underlying diagnosis in about 0.5% of patients with hypertrophic cardiomyopathy (which carries a prevalence of 1 in 300 in the adult population) [2,3].

Patients with classic FD have absent or very low α-GAL activity [4], early onset of symptoms, and progressive multisystemic involvement. In comparison, atypical FD or the cardiac variant have some residual or lower than normal α-GAL activity (6), present at a later age, and have variable onset and predominantly only cardiac involvement, as opposed to a multi-system involvement. In heterozygous females, α-GAL activity may be low-normal or variably deficient. These patients also have variable onset, and their systemic manifestations depend on the underlying GLA mutation, the pattern of organ-specific X-chromosome inactivation, and residual enzyme activity [5,6].

## 2. Pathophysiology

The deficiency of α-GAL activity impairs globotriaosylceramide (GL3) metabolism, leading to the progressive accumulation of GL3 and lyso-GL3 (globotriaosylsphingosine) in various organs, especially the heart, kidneys, and the cerebrovascular system [7]. Thurberg et al. [8] demonstrated that GL3 is deposited as dense, beaded cytoplasmic inclusions within the interstitial capillary endothelial cells and cardiomyocytes on the endomyocardial biopsies. These inclusions appear as laminated myelin figures on an electron microscope. As the disease progresses, inclusions grow larger to form clusters that may protrude into the capillary lumen. GL3 accumulation in the microvasculature results in endothelial injury, intima-media thickening, and atheroma production.

GL3 and lyso-GL3 accumulation induce cytokine production, coagulation activation, and oxidative stress [8]. This results in tyrosine nitration, DNA damage, and apoptosis. Lyso-GL3 binds to toll-like receptor 4, activating nuclear factor κB and T lymphocytes to initiate cytokine production [9]. TNF-α (tumor necrosis factor), TNF receptor (TNFR1 and TNFR2), and IL-6 (interleukin) are significantly elevated in FD patients compared to healthy cohorts, indicating the major role of chronic inflammation in the pathogenesis of FD. TNFR2, TNF-α, and IL-6 are significantly elevated in FD patients with left ventricular hypertrophy (LVH) than those without LVH (*p* = 0.045, *p* = 0.024, *p* = 0.001, respectively). TNFR1 and TNFR2 levels increase with worsening myocardial fibrosis (*p* = 0.014, *p* = 0.014, respectively) and renal dysfunction (*p* < 0.001, *p* < 0.001, respectively) compared to patients without significant fibrosis or chronic renal disease [9]. TNF-α, TNFR1, and TNFR2 levels are higher in patients with severe heart failure compared to healthy subjects [10,11], and increased levels predict 24-month mortality (χ^2^ = 14.5, χ^2^ = 26.1 and χ^2^ = 15.1, respectively, all *p* ≤ 0.0001) [12].

Significant GL3 accumulation in cardiomyocytes can be detected as early as childhood and adolescence [13]. Cardiomyopathy develops as a result of the progressive accumulation of GL3 in myocytes, endothelium, smooth muscle cells, fibroblasts, and conductive tissue. LVH is present in 50% of males and 33% of females with FD [1]. LVH and diastolic dysfunction may occur in the early stages of the disease. However, in females, diastolic dysfunction and myocardial fibrosis can develop without LVH [14]. Untreated FD cardiomyopathy progresses to heart failure and can be accompanied by myocardial ischemia, valvular dysfunction, and life-threatening arrhythmias. Severe microvascular dysfunction is the primary mechanism for myocardial ischemia and angina in these patients in the absence of epicardial coronary artery disease.

## 3. Diagnostic Evaluation

FD is a progressive, multisystemic disease requiring early clinical recognition by clinicians to avoid life-threatening complications of renal failure, heart failure, arrhythmias, and stroke. Missed diagnoses are common in FD patients and may be initially misdiagnosed as rheumatological diseases (39%), arthropathy (15%), neuropsychological disease (13%), or fibromyalgia syndrome (7%) [15]. Early cardiac red flags include left ventricular wall thickness (40.2%), diastolic dysfunction (27.8%), systolic dysfunction (6.7%), conduction abnormalities (P wave or PR shortening [early disease, 14%], bradycardia [72%], chronotropic incompetence, and atrioventricular blocks [advance disease, 14.8%]), and valvular heart disease (mitral [57%] and aortic valve thickening [47%]). Extra-cardiac red flags during the 1st and 2nd decades of life include cornea verticillata, angiokeratomas, hypohidrosis, recurrent diarrhea, and abdominal and neuropathic pain. Hearing loss, tinnitus, vertigo, cryptogenic stroke, and renal dysfunction are common in the 3rd and 4th decades of life [7]. A comprehensive diagnostic approach is needed for early diagnosis, including the early recognition of clinical red flags, and appropriate genetic and/or enzymatic testing and imaging tests depending on the organ system involved. Common tests include electrocardiogram, echocardiogram, and biomarkers (plasma and urinary GL3 and lyso-GL3, eGFR, proteinuria, B-type natriuretic peptide, troponin I/T). Depending on symptoms and clinical suspicion, patients may require an MRI of the brain, renal ultrasound, and other targeted testing (e.g., pulmonary function tests, audiogram, etc.). Other novel tests are available and are primarily used for disease monitoring and progression in the research arena, such as serum levels of matrix metalloproteinases, galectin-1/3, tumor necrosis factor-α, TNF receptors, and interleukin-6. Plasma biomarkers can increase sensitivity for preclinical disease and help initiate early treatment in children, atypical FD, and heterozygous females with polymorphism.

It is important to differentiate FD cardiomyopathy from other forms of unexplained LVH, especially in the absence of extra-cardiac manifestations in atypical phenotypes and heterozygote females. The diagnosis of FD is confirmed by enzyme activity assay and/or genetic testing following high clinical suspicion or cascade familial screening. A tissue biopsy is rarely required. In most US centers, genetic testing is the initial screening test due to its wide availability.

Multimodality imaging is essential in recognizing early cardiac involvement. Cardiac magnetic resonance imaging (CMR) provides a more accurate structural and functional evaluation than transthoracic echocardiography (TTE). Additionally, CMR can perform tissue characterization using late gadolinium enhancement (LGE) and novel parametric mapping techniques such as native T1 mapping and extracellular volume (ECV) measurement. A consensus statement by the Society for Cardiovascular Magnetic Resonance (SCMR) [16] recommends parametric mapping in evaluating myocardial disease. The early recognition of myocardial involvement can significantly impact decision-making in asymptomatic disease by allowing better risk stratification and therapy targeting. However, CMR is less readily available than TTE, and its use is limited due to the need for an experienced technician and interpreter. The role of CMR and TTE in structural and functional evaluation, tissue characterization, and monitoring the response to therapy is summarized in Table 1.

## 4. Patient Selection and Treatment

The early recognition of organ involvement, especially heart, kidneys, and the cerebrovascular system, is essential to initiate therapy promptly and prevent potentially irreversible complications. Cardiac and renal involvement may not manifest clinically until adolescence or adulthood. Unexplained LVH in the absence of extracardiac manifestation requires high clinical suspicion and community awareness to distinguish FD cardiomyopathy from other etiologies of hypertrophic myocardium. Renal damage is also typically subclinical in its early stages and requires a biopsy for identification. Children with FD mutations should be treated as soon as the symptoms develop. However, boys with a classic FD mutation should be treated as early as 8–10 years of age, even without symptoms [45]. The European Fabry Working Group consensus statement recommends the initiation of therapy in both classic and non-classic FD patients of both sexes when there is an increased LV wall thickening >12 mm (Class 1 recommendation) [46].

### 4.1. Existing Therapies

**Figure 1:**
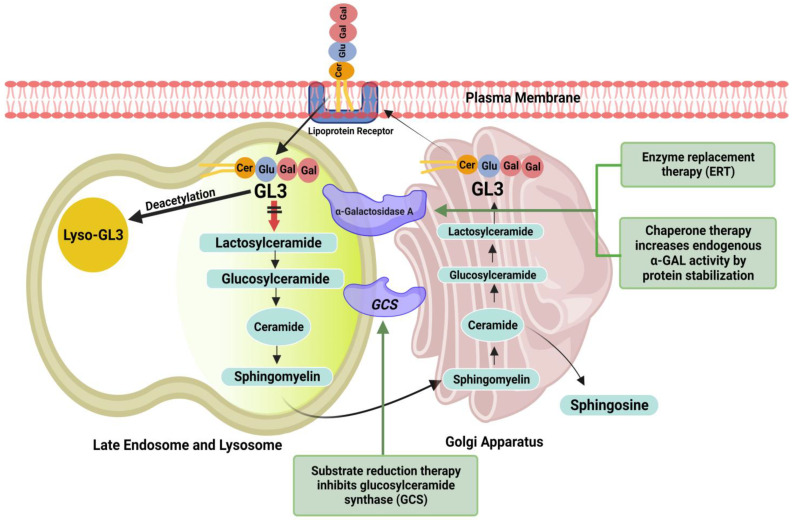
Overview of the ceramide pathway and therapeutic targets. ERT replaces deficient endogenous enzyme with recombinant enzyme (Agalsidase-α or Agalsidase-β). Chaperone (migalastat) binds reversibly to and stabilizes amenable gene variants of α-GAL, thereby facilitating proper trafficking of the enzyme to lysosomes and increasing enzyme activity. Substrate reduction therapy (lucerastat or venglustat) causes inhibition of glucosylceramide synthesis and reduces the accumulation of glycosphingolipids, including glucosylceramide and globotriaosylceramide (GL3).

The planning and initiation of FD treatment should be performed at an interdisciplinary FD center. The main therapeutic goals are symptom reduction to improve quality of life, preventing multiorgan involvement, and halting disease progression to improve life expectancy. Established treatment options to reduce GL3 accumulation inside lysosomes include replacing deficient α-GAL endogenous enzyme with recombinant ERT and increasing α-GAL enzyme availability inside lysosomes by correcting the misfolding of α-GAL through chaperone therapy (Figure 1). Table 2 summarizes the mechanism of action and physiological effects of available and investigational drugs for treating FD.

#### 4.1.1. Enzyme Replacement Therapy

ERT has been available as a treatment option for FD since 2001 [47,48]. Brady et al. [49] made the first attempt in 1973 to administer the α-GAL to two FD patients, purified from human placental tissue. The infusion was well tolerated and improved enzyme activity by 68%. The purification of α-GAL from the placenta and spleen was complex and insufficiently available; advances in biotechnology enabled the production of recombinant α-GAL. Two preparations, agalsidase-β and agalsidase-α are currently approved as life-long options for FD treatment.

The European Fabry working group consensus document [46] recommendations for the initiation and discontinuation of ERT and contraindications are summarized in Table 3.

Nordin et al. [35] demonstrated that after 12 months of ERT, LVH-positive patients had a detectable, small reduction in LVMi (117 ± 38 versus 114 ± 36 g/m^2^; *p* = 0.048) and native T1 lowering (partial normalization; 920 ± 48 ms vs. 902 ± 47; *p* = 0.008). However, in LVH-negative patients, who were all females, the reduction in native T1 lowering was not statistically significant (940 ± 46 vs. 948 ± 60 ms; *p* = 0.480). Overall, 83% had an increase in native T1 value after one year of ERT. There was no significant change in ECV and GLS in both LVH-positive and LVH-negative groups.

Koeppe et al. [50] observed a significant decrease in end-diastolic wall thickness and a decline in hypokinesia after 12 months of ERT in LGE-negative patients.

Wuest et al. [39] followed FD patients for 13 ± 1 months after ERT; there was a significant reduction in LV and RV mass, LV and RV EDV, and LV ESV, while LVEF increased significantly. There was no significant change in RV ESV, SV, and EF.

##### Agalsidase-β

Agalsidase-β (Fabrazyme, Sanofi Genzyme) is produced in Chinese hamster ovary cells and is available as an intravenous infusion every two weeks, with a recommended dose of 1.0 mg/kg body weight.

Arends et al. [51] compared the effects of agalsidase-α and agalsidase-β at their registered doses. FD patients treated with agalsidase beta, especially men with classical FD, had a more significant decrease in plasma lyso-GL3 (β: −18 nmol/L, *p* < 0.001), and LV mass index (LVMI, OR 2.27, *p* = 0.03), while eGFR slopes and event rate (HR 0.96, *p* = 0.87) were similar for both enzymes. The risk of developing antibodies was higher with agalsidase beta (OR 2.8, *p* = 0.04). However, its effect continued in the presence of antibodies.

Banikazemi et al. [52] studied the effect of agalsidase-β therapy in patients with advanced FD. Agalsidase beta delayed the time to the first clinical event (renal, cardiac, or cerebrovascular event or death) compared to the placebo group after adjusting for baseline proteinuria (hazard ratio 0.47, *p* = 0.06). The study also showed that the clinical benefit was greater with early initiation of treatment before the onset of irreversible organ damage.

Wraith et al. [53] demonstrated the safety and efficacy of agalsidase-β (1 mg/kg) in pediatric patients. Fourteen male and two female patients, 8 to 16 years old, were followed for 48 weeks. GL-3 was cleared from the dermal capillary endothelial cells, and gastrointestinal symptoms declined. The treatment was well tolerated, and adverse events were mild to moderate infusion-associated reactions. 

Weidemann et al. [38] noted a statistically significant 28% decrease in LV inferolateral wall thickness and a 10% decrease in LV mass by CMR in patients treated in an open-label study with 1.0 mg/kg body weight of agalsidase-β for 12 months. The peak systolic strain rate and end-systolic strain also increased significantly in the posterior wall. Both radial and longitudinal strains showed improvement.

Imbriaco et al. [43] evaluated FD patients treated with agalsidase-β at 1 mg/kg every other week (study 1) and after a mean treatment duration of 45 months (study 2); LV mass (188 g vs. 153 g) and LV wall thickness (16 mm vs. 14 mm) reduced significantly. Furthermore, a significant reduction in myocardial T2 relaxation times was noted in all myocardial regions (interventricular septum 80 ms versus 66 ms, apex 79 ms versus 64 ms, and lateral wall 80 ms versus 65 ms). Changes in LV ejection fraction were not significant. All patients showed improvement in symptoms.

Messalli et al. [44] evaluated FD patients with CMR after 48 months of treatment with agalsidase-β, and a significant reduction was observed in LV mass, wall thickness, and native T2 values. There was no significant change in LVEF.

Nowak et al. [54] performed a prospective observational study on 14 classic FD patients switched from agalsidase-α to agalsidase-β at the respective licensed doses. After the switch, there was a significant reduction in plasma lyso-GL3 levels (mean reduction of 30.1%; *p* = 0.004). Plasma lyso-GL3 reduction correlated negatively with the residual α-GAL activity in male patients (r = −0.803; *p* = 0.009). The results demonstrated that agalsidase-β is significantly more effective than agalsidase-α and should be used as first-line therapy in classic FD males with no residual enzyme activity.

##### Agalsidase-α

Agalsidase-α (Replagal, Takeda) is produced in a human cell line (human fibrosarcoma cells HT-1080) and is available as an intravenous infusion every two weeks at the recommended dosage of 0.2 mg/kg body weight.

Kampmann et al. [55] studied the effectiveness of agalsidase-α enzyme replacement in FD with a 10-year treatment follow-up. HF classification improved by at least 1 class in 52%, and stabilized or improved angina scores in 98%; no patients without LVH developed LVH, renal function was generally maintained, and in patients with LVH, improvements were apparent after 1 year, with benefits in male patients being sustained after 10 years and deterioration being controlled in female patients.

Hughes et al. [37] followed FD patients after treatment with agalsidase-α by CMR and TTE and noted the regression of LVH due to the progressive clearance of GL3 content from cardiomyocytes.

Schiffmann et al. [56] performed a 6.5 year open-label follow-up study in pediatric patients (median age: 10.8 [8.6–17.3] years; 10 [90.9%] males) treated with agalsidase-α. ERT was well tolerated and had a stabilizing effect. Plasma and urinary GL3 reductions were maintained. LVMI and eGFR remained within a normal range, and heart rate variations improved.

Tsuboi et al. [57], in an observational study of 11 FD patients, demonstrated that it is safe to switch from agalsidase-β to agalsidase-α without the loss of efficacy and that it is a feasible option in patients with an infusion-related allergic reaction to agalsidase-β.

##### Anti-Agalsidase Antibody

Various studies have identified the anti-agalsidase antibodies (ABs) and investigated their significance and effect on enzyme activity. Mauhin et al. [58] studied the ABs characterization in depth. IgG ABs were present in 40% of the FD men exposed to agalsidase (α 30.8%, β 44.4%, α and β 42.9%). AB presence was independent of the duration of exposure (odds ratio (OR) = 1.1, *p* = 0.09). ABs were more prevalent in classic FD than other phenotypes (58.6% vs. 6.7%, *p* = 0.0005). Mutations leading to truncated α-GAL proteins (deletions, nonsense, and frameshift mutations) were more frequently associated with ABs than missense mutations (55.6% vs. 12.0%, *p* = 0.006). IgG1 AB was the most frequently observed antibody in men (89%), but the highest concentrations were found for IgG4. IgG ABs developed in only 8% of FD women after 1.8 and 10.8 months of exposure to agalsidase-α and -β, respectively. The lyso-GL3 levels were higher in the AB-positive patients (median 33.2 ng/mL vs. 12.5 ng/mL, *p* = 0.005). There was a clear correlation between inhibition and AB titers. However, despite the inhibiting effect, ABs had no obvious clinical impact, and their association with lyso-GL3 levels could be a hallmark of the severity in classic FD.

Lenders et al. [59] evaluated the serum-mediated inhibition of ERT. Inhibition was noted in 40% of agalsidase-treated males, regardless of the type of ERT (α or β). On a longitudinal 5-year retrospective analysis, agalsidase inhibition was associated with higher lyso-GL3 levels, worse disease severity scores, more FD-related symptoms, greater LVM (*p* = 0.02), and lower renal function (*p* = 0.04).

Rombach et al. [60] demonstrated that 58% of males developed antibodies against agalsidase and persisted for up to 10 years of ERT. No females developed AB. AB presence was associated with a less robust decrease in plasma lyso-GL3 and had a negative effect on urinary GL3 reduction.

#### 4.1.2. Chaperone Therapy

An oral pharmacologic chaperone binds to and stabilizes amenable gene variants of α-GAL, thereby facilitating proper trafficking of the enzyme to lysosomes and increasing enzyme activity (Figure 1).

##### Migalastat

Migalastat is the first-in-class long-term oral option for FD patients with amenable GLA variants. The data from clinical trials demonstrate its safety and effectiveness. The amenable mutation data can be found on the Galafold website [61].

ATTRACT, a phase III clinical trial (NCT01218659), demonstrated the promising role of oral migalastat monotherapy as an alternative treatment option to intravenous ERT for FD patients with amenable mutations [62]. Migalastat and ERT had similar effects on renal function and a significant reduction in LVMI with migalastat treatment (−6.6 g/m^2^ (−11.0 to −2.2)) but no significant change with ERT. Renal, cardiac, and cerebrovascular events were lower in patients on migalastat than ERT (29% vs. 44%, respectively). In an open-label extension of the ATTRACT study, Feldt-Rasmussen et al. [63] showed that migalastat was well tolerated and reduced LVMI at 36 months. Another phase III, randomized, double-blind FACETS trial (NCT00925301) demonstrated similar results in ERT-naive patients with the classic phenotype and amenable GLA variants. There was a reduction in LVM and a stabilization of renal function for up to 24 months with migalastat, regardless of disease severity [64].

Landers et al. [65] analyzed outcomes in 54 patients treated with migalastat from a prospective observational multicenter study (FAMOUS). The treatment with migalastat was safe and reduced LVMI after 24 months (all: −7.5 ± 17.4 g/m^2^, *p* = 0.0118; females: −4.6 ± 9.1 g/m^2^, *p* = 0.0554; males: −9.9 ± 22.2 g/m^2^, *p* = 0.0699).

Hughes et al. [66] evaluated the long-term multisystemic efficacy and safety of migalastat in 97 FD patients with an amenable GLA variant (48 ERT naive; 49 ERT experienced) who were enrolled in Phase III clinical trials (ATTRACT and FACETS). The migalastat cohort had a lower eGFR, higher age, and higher LVMI at the beginning of migalastat treatment compared with the ERT cohort. Migalastat was associated with a lower incidence of Fabry-associated clinical events (FACEs; renal, cardiac, and cerebrovascular events) for both ERT-naive and ERT-experienced patients receiving migalastat treatment for up to 8.6 years (48.3 events per 1000 patient-years overall). Baseline eGFR was a significant predictor of FACEs. The early initiation of treatment and preserving renal function are vital for improving outcomes.

### 4.2. Emerging Therapies

Emerging therapies include plant-derived ERTs, substate reduction, and gene therapy.

#### 4.2.1. Next Generation ERT

##### Pegunigalsidase-α

Plant-derived forms of ERT were designed to increase plasma half-life and reduce immunogenicity, thereby enhancing efficacy. In a study by Schiffmann et al. [67], 16 patients were followed for 1 year of treatment. The mean plasma half-life ranged from 53 to 121 hours. All patients with classic FD (11 male and 1 of 7 female patients) had an 84% reduction in the renal peritubular capillary GL3 inclusions, and the mean estimated glomerular filtration rate remained stable. Three patients developed treatment-induced IgG anti-drug antibodies (ADAs), and all became ADA-negative after one year of treatment. 

##### Moss α-GAL

It is the moss-derived form of α-GAL currently undergoing phase II and III clinical trials. Physcomitrella patens is a genetically modified moss. Hennermann et al. [68], in a phase I trial, showed that moss α-GAL is safe and leads to a prolonged reduction of GL3. It is taken up by the mannose receptor, which is expressed on macrophages, endothelial cells, and kidney cells.

#### 4.2.2. Substrate Reduction

Substrate reduction therapy focusses on the inhibition of glucosylceramide synthesis with a subsequent reduction of GL3 accumulation in the cells:

##### Lucerastat

Guerard et al. [69] demonstrated that Lucerastat is well tolerated in patients with Fabry disease over 12 weeks. A marked decrease in plasma GL3 was observed, suggesting clinical potential for Lucerastat in patients with FD.

##### Venglustat

It is an oral glucosylceramide synthase (GCS) inhibitor to reduce the production of glucosylceramide (GL1), which subsequently decreases GL3 production and accumulation in cells. Peterschmitt et al. [70] conducted a phase I clinical trial on healthy volunteers and demonstrated a favorable safety and tolerability profile.

Deegan et al. [71], in a phase 2a clinical trial and its extension study, demonstrated that there are no biochemical or histological indications of the progression of FD over 3 years of follow-up.

##### Genz-682452

It is a novel, orally available GCS inhibitor with pharmacological and safety profiles with the potential for treating FD. Ashe et al. [72] demonstrated the reduced tissue levels of GL3 and lyso-GL3 in mice. Genz-682452 can traverse the blood-brain barrier and reduce glycosphingolipid accumulation in the brain. Study results also demonstrated that combining substrate reduction with ERT may increase the therapeutic benefits in Fabry disease.

#### 4.2.3. Gene Therapies

**Figure 2:**
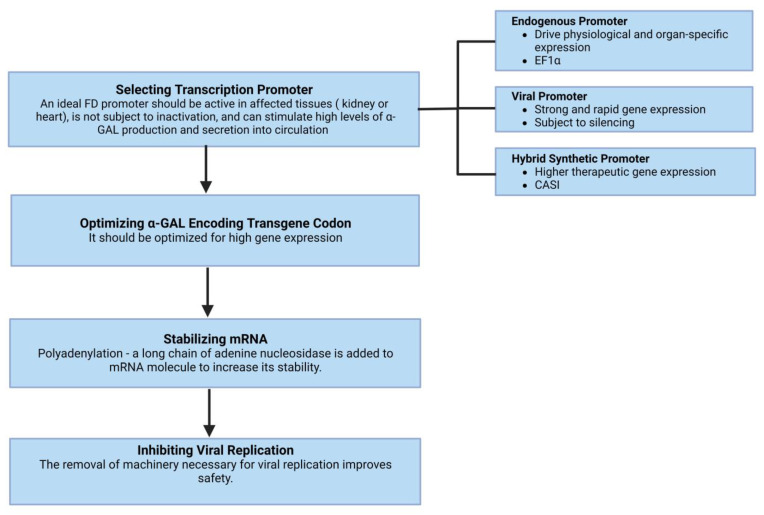
The basic requisites of effective gene therapy include a transcription promoter that is active in affected tissues and optimized for high gene expression. Other essential components include the stabilization of mRNA and inhibiting viral replication.

Gene therapies are being developed as a long-term treatment option based on the hypothesis that the targeted cells will overexpress α-GAL, secrete it, and subsequently, other cells will uptake it via mannose-6-phosphate receptors and transport it to the lysosomes. Effective gene therapy requires a transcription promoter active in affected tissues and is optimized for high gene expression, stabilization of mRNA, and inhibiting viral replication (Figure 2). More than two decades ago, Takenka et al. [73] demonstrated that α-GAL was overexpressed and secreted into circulation by the transduced bone marrow cells and remained stable over a significant time. In a study by Medin et al. [74], transduced cells showed a greater than 16-fold increase in enzyme activity and cross-corrected unmodified FD cells. The duration of cross-correction depends on the type of the targeted cells; hematopoietic stem cells [75] or liver hepatocytes with prolonged lifespans can lead to prolonged enzyme expression.

**Figure 3:**
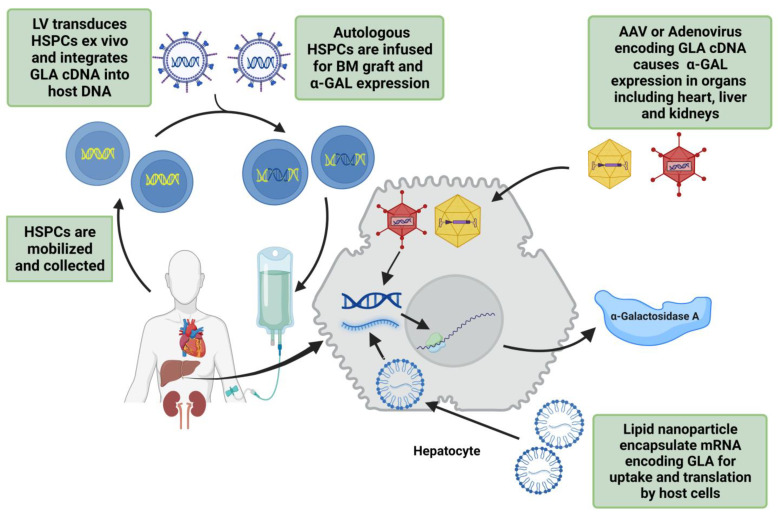
Overview of the gene therapy techniques being used in trials for FD treatment. The first step is to mobilize hematopoietic stem/progenitor cells (HSPCs) and integrate GLA cDNA into host DNA using lentivirus (LV). These transduced HSPCs are transplanted back into bone marrow for α-galactosidase A (α-GAL) expression. Adeno-associated virus (AAV) or adenovirus can also be used for GLA cDNA transfer into target cells. Lipid nanoparticle encapsulated mRNA encoding human GLA is another safe method of endogenous α-GAL expression.

A possible disadvantage of currently investigated gene therapy approaches for FD is that they lack target specific vectors or mRNA/cDNA for cardiac, renal, and cardiovascular tissues. Current methods are similar to ERT in that they need systemic administration or to be targeted to liver or HSPCs, thus their effectiveness relies on the overexpression of α-GAL into circulation for other tissues to uptake. However, if gene therapy does become available, it has the advantage of long-term supraphysiologic enzyme expression and a lack of anti-α-GAL antibodies. Figure 3 gives an overview of various gene therapy techniques.

##### Lentivirus

Lentivirus (LV)-based strategies insert GLA cDNA into the genomic DNA of hematopoietic stem/progenitor cells (HSPCs). The expression of α-GAL is for the lifetime of transduced HSPC and its progeny. Clinical trials addressing other diseases, such as β-thalassemia, have shown long-term safety [76].

Yoshimitsu et al. [77] demonstrated a two-fold increase in α-GAL activity in Fabry mice compared to wild-type controls and a reduction of GL3 in all organs after the transplantation of LV-transduced bone marrow mononuclear cells (BMMNCs). The transduction of mobilized peripheral blood CD34^+^ cells from a Fabry patient also appropriately increased enzymatic activity. This study showed the sustained correction for FD after a single LV-mediated transduction of hematopoietic cells.

Pacienza et al. [78] studied the transplantation of normal human CD34(+) cells transduced with LV in congenic nonobese diabetic (NOD)/severe combined immunodeficiency (SCID)/Fabry mice. Only the therapeutic group showed a significant increase in plasma α-GAL activity and a reduction in GL3 in the heart and kidneys.

In the first-in-human phase 1 clinical trial (NCT02800070) by Khan et al. [79], five adult males with classical FD were infused with autologous LV-transduced, CD34^+^-selected, HSPCs engineered to express α-GAL. No serious adverse events are reported. All patients produced α-GAL to near normal levels within one week, and reductions in plasma and urine GL3 and lyso-GL3 were noted. The study is estimated to complete in February 2024.

Another multinational phase I/II clinical trial (NCT03454893) is ongoing to assess the efficacy and safety of ex-vivo, LV-mediated gene therapy AVR-RD-01 for treatment-naive patients with classic FD.

##### Adenovirus and Adeno-Associated Virus

Adenovirus has been used in various preclinical models to treat FD. It can transduce various cell types and has prolonged transgene expression. Ziegler et al. [80] demonstrated successful α-GAL expression in Fabry mice by adenovirus-mediated gene transfer. The α-GAL levels were elevated in the liver, kidney, lung, and spleen within 3 days and lasted 12 weeks; however, dose-dependent liver toxicity was also noted. A lower dose of adenovirus improved toxicity while maintaining GL3 clearance when Kupffer cells were depleted, suggesting the use of strategies that reduce adenovirus interaction with the reticuloendothelial system (RES) and pretreatment of mice with gamma globulins [81]. The toxicity noted in these preclinical trials, as well as reports of hepatic lesions, neutropenia, and thrombocytopenia during studies for oncolytic purposes [82], have caused researchers to switch to the adeno-associated virus (AAV).

The long-term safety and prolonged gene expression with AAV-mediated gene transfer have been observed in various clinical trials of other diseases, such as hemophilia B [83,84]. AAV is capable of endogenous α-GAL expression within targeted disease phenotype cells in the heart [85] and liver for expression and systemic uptake. Various AAV capsids have been engineered to target other tissues but have not been applied to FD yet.

Jung et al. [86] constructed a recombinant AAV vector encoding human α-GAL and injected it into the hepatic portal vein of the Fabry mice. The enzymatic activity increased by 20–35% in two weeks compared to normal mice, and the high levels were maintained for as long as 6 months after treatment in the liver and other organs. GL3 levels reduced to near normal at 2 and 5 weeks posttreatment, and the effect continued in the liver, spleen, and heart for up to 25 weeks with no significant immune response to the virus or α-GAL.

In another study by Takahashi [87], an AAV vector containing the α-GAL gene was injected into the quadriceps muscles of Fabry knockout mice. It resulted in a 25% increase in plasma α-GAL activity for at least 30 weeks without the development of anti-α-GAL antibodies. GL3 was completely cleared by 25 weeks, and echocardiography showed improved LVH.

Park et al. [88] studied the effects of a single injection of recombinant AAV encoding the human α-GAL gene to Fabry mice using a modified chicken β-actin (CAG) as a promoter. It resulted in the stable expression of α-GAL for more than 6 months and a significant reduction in GL3 levels in the liver, heart, spleen, kidney, lung, and small intestine.

The first-in-human phase I/II clinical trial (NCT04046224) is ongoing to evaluate the safety and tolerability of ST-920, a recombinant AAV2/6 vector encoding the cDNA for human α-GAL. Other ongoing clinical trials include MARVEL 1 (NCT04040049) and NCT04455230 to assess the long-term safety and efficacy of FLT190 (AAVS3).

A major challenge with AAV-mediated gene transfer is the eventual decline in expression due to episomal AAV clearance or dilution through cell division. Another major challenge is to overcome anti-AAV antibodies that will hamper re-administration. The strategies for re-administration to minimize or avoid immunity against AAV include alternate delivery site [89] mutant capsid design [90], the addition of the empty capsid to the final vector formulation [91], altered vector doses [84], transient immunosuppression [92], using an IgG degrading enzyme [93], or the elimination of transfer of capsid-expressing DNA [94].

##### Plasmid DNA and mRNA

The DNA and RNA-based delivery of the GLA gene has long-term safety and is capable of endogenous α-GAL expression within targeted tissues without causing immunogenicity [95]. Zhu et al. [96] and DeRosa et al. [97] demonstrated preclinical evidence for the systemic use of lipid nanoparticle encapsulated mRNA encoding human GLA for treating FD. The administration of α-GAL mRNA to GLA-deficient mice showed dose-dependent enzyme activity and GL3 and lyso-GL3 reduction. Safety and translatability were confirmed by multiple administrations to non-human primates.

The demonstrated safety of mRNA encoding human GLA to express α-GAL makes it a promising treatment option for FD. However, repeated administration is required due to the transient half-life of mRNA and its inability to correct the disorder at the genomic level.

##### CRISPR (clustered regularly interspaced palindromic repeats)/Cas (CRISPR-associated genes)

CRISPR/Cas can perform targeted DNA breaks for gene editing [98]. DNA breaks trigger DNA repair pathways through homology-directed repair (HDR) or non-homologous end joining (NHEJ) [99,100]. This strategy can be applied in treating FD by targeting dividing cells with HDR, or non-dividing cells, such as HSPCs, with NHEJ-based insertion or HDR followed by enrichment. The main hurdle will be low transgene insertion rates. CRISPR/Cas relies on programmable guide RNAs (gRNA) to target effector Cas proteins and cause double-stranded breaks in the genome for gene insertion or silencing. 

Current CRISPR-based gene-editing tools include gene insertion with HDR, base editing, CRISPR/Cas fused transposase and prime editing. Chang et al. [101] used CRISPR/cas9 with dual gRNAs to delete the mutation (GLA IVS4 + 919 G > A) related to aberrant GLA splicing in the cardiac FD phenotype. It significantly increased α-GAL enzyme activity and cleared GL3 in FD fibroblasts, thus proving a feasible approach for treating cardiac variant FD.

The main challenge with gene editing is achieving high enough gene insertion rates in the appropriate cell types to correct a disease phenotype and restore organ function. Safety is also a significant concern with the gene-editing strategy. It can make potentially unwanted genomic changes on-target or genomic alterations at unwanted loci, called “off-targets,” and lead to genotoxic effects. Various advancements have been made to improve gRNA design for more precise DNA targeting, high-fidelity nucleases to avoid off-target cutting, Cas nuclease silencing to prevent unwanted DNA cutting, and tissue-specific targeting through AAV or nanoparticles. Furthermore, gene-editing therapies are costly, as high as 1 million USD or more. Gene-editing technology may also require the optimization of gRNA for various populations with different genotypes, thus further increasing the cost. However, if gene editing becomes available for FD, it could offer a long-term “cure”.

### 4.3. Heart Transplantation

Early recognition of cardiac involvement in FD is essential to start early treatment and stop the disease progression to prevent life-threatening complications. Misdiagnoses and significant diagnostic delays are common in FD, especially in female patients resulting in end-stage heart failure. Heart transplantation should be offered to selected patients. The ERT is mandatory after a heart transplant to avoid the FD effect on the donor graft [102].

## 5. Prognosis

Misdiagnoses are common in FD, with significant diagnostic delays in females (16.3 ± 14.7 years) and males (13.7 ± 12.9) [5,15], resulting in the late initiation of treatment and a worse prognosis. FD can significantly reduce life expectancy, approximately by 20 years in males and 15 years in females [103,104]. Renal involvement and left ventricular hypertrophy are the main determinants of major cardiac and non-cardiac events [22]. Cardiac disease is the major cause of death in FD. The early initiation of treatment is imperative to avoid multiorgan involvement and disease progression. The efficacy of treatment decreases with advancing stages of cardiomyopathy, thus worsening the overall prognosis [7]. Cardiomyopathy is the leading cause of death in men (34%) and women (57%) with FD [105]. A prognostic model developed by Orsborne et al. [106] provides an accurate estimate of the 5-year risk of adverse cardiac outcomes. This model is based on the CMR-derived native myocardial T_1_ dispersion and left ventricular mass index (LVMi), in addition to age. T_1_ mapping is a superior technique to detect GL3 accumulation and diffuse fibrosis/inflammation, and LVMi is an independent predictor of adverse cardiac events in FD [107]. The long-term prognosis is also affected by clinical indices of extracardiac involvement, such as renal function, proteinuria, and neurological dysfunction.

## 6. Conclusions

The timely initiation of treatment is crucial in FD to avoid multi-organ involvement and halt life-threatening complications of cardiomyopathy and nephropathy that can significantly reduce life expectancy. Established treatment options include replacing deficient endogenous α-GAL with recombinant ERT (agalsidase-α and agalsidase-β) or increasing α-GAL enzyme activity inside lysosomes by chaperone therapy (migalastat). Next-generation plant-derived forms of ERT (pegunigalsidase-α and moss α-GAL) are promising, with an increased plasma half-life and reduced immunogenicity. Other emerging therapies include substrate reduction (Lucerastat and venglustat) and gene therapy. Gene therapy has the potential for a long-term cure and tissue-directed treatment – emerging data are promising, but further research is required to establish the clinical efficacy and safety.

## Figures and Tables

**Table 1:** The role of multimodality imaging in evaluating FD cardiomyopathy and response to treatment. Left ventricle (LV), right ventricle (RV), left ventricular hypertrophy (LVH), left ventricular mass (LVM), right ventricular hypertrophy (RVH), tissue doppler imaging (TDI), early diastolic mitral inflow velocity (E), basal septal mitral annular tissue velocity (e’), global longitudinal strain (GLS), late gadolinium enhancement (LGE), global longitudinal strain (GLS), circumferential strain (CS), global radial strain (GRS), globotriaosylceramide (GL3), extracellular volume (ECV).

Structural Evaluation	Functional Evaluation			Tissue Characterization		
Ventricles and Atria	Diastolic Dysfunction	Systolic Dysfunction	Tissue Doppler Imaging Strain Analysis	LGE Pattern	T1 Mapping Extracellular Volume	T2 Mapping
The majority of the patients have concentric LV wall thickening [17]. Papillary muscle hypertrophy can contribute to the LV mass and cause mid-ventricular obstruction [18,19]. RV involvement is present in 31–71% of the patients, causing RV hypertrophy with normal chamber size [20,21]. RVH and RV systolic function show significant association with clinical events, but RV involvement does not influence prognosis [22]. Atrial dilatation is more common in patients with LVH and fibrosis [23,24], and left atrial dysfunction is associated with Bradyarrhythmia [25].	LV diastolic dysfunction may occur before the development of LVH [26]. RV diastolic dysfunction can develop with advancing left ventricular cardiomyopathy.	LV systolic dysfunction does not usually occur until the late stages of cardiac involvement. Despite RV hypertrophy and fibrosis, RV systolic function is generally preserved.	TDI is an accurate and sensitive tool for diagnosing myocardial dysfunction, even before the development of LVH [24,27]. Septal E/e’ can predict replacement fibrosis in the absence of LVH and diastolic dysfunction [28]. GLS and GRS are significantly reduced, and GLS impairment correlates with GL3/lyso-GL3 elevation, thus having a potential role in detecting early cardiac involvement [29,30]. Loss of base-to-apex CS gradient is also an early marker of cardiac involvement [31]. Longitudinal systolic strain, systolic strain rate, and longitudinal early diastolic strain rate are superior TTE measures of LV function and are reduced independent of LVH [32]. Reduced longitudinal strain in the basal inferolateral wall can help differentiate from other hypertrophic cardiomyopathies [23].	Basal to mid inferolateral mid-myocardium [33].	Initially, native T1 values are reduced, but later there is pseudonormalization in the areas of LGE. It can reliably distinguish FD from other causes of LVH (27). ECV is normal [34] but may increase in the area of LGE as a biomarker for fibrosis.	T2 values are elevated in the area of LGE, indicating chronic inflammation [35,36].
Monitoring of treatment efficacy						
Ventricles and Atria	Diastolic dysfunction	Systolic dysfunction	Tissue doppler imaging Strain analysis	LGE pattern	T1 mapping Extracellular volume	T2 mapping
LVM regression occurs in patients with baseline LVH. Patients with little or no LGE at baseline also showed a decrease in LVM [35,37,38]. LVM reduction varies among various studies (10–27%), likely depending on the timing of therapy, the intensity of therapy, the stage of cardiomyopathy, and other confounding factors, such as age, sex, hypertension, etc. RV mass may decrease with ERT [39].	No change [38] or very minor improvement in diastolic dysfunction is noted with ERT [40].	Radial and longitudinal function of LV improves after 12 months of ERT, especially in the last 6 months of treatment [38]. Patients without fibrosis showed improvement in systolic function over 3 years on ERT, although no improvement was noted in patients with mild or severe fibrosis [41].	Significant increase of peak systolic strain rate (radial function, baseline, 2.8 ± 0.2 s^−1^; 12 months, 3.7 ± 0.3 s^−1^; *p* < 0.05) and end-systolic strain (baseline, 34 ± 3%; 12 months, 45 ± 4%; *p* < 0.05) is noted in the posterior wall after 12 months of ERT [38]. Patients without fibrosis showed improvement in systolic radial strain rate (2.3 ± 0.4 to 2.9 ± 0.6 s^−1^; *p* = 0.045) over a long-term follow-up of 3 years on ERT. No improvement in patients with mild or severe fibrosis [41]. LV inferolateral regional strain can predict the progression of LGE [42].	No improvement is noted with treatment.	Reduction in T1 lowering with ERT [35].	Decrease in T2 due to a reduction in myocardial lipid burden [43,44].

**Table 2:** Available and investigational drugs for the treatment of FD with their mechanism of action, route of administration, and physiological effects. α-galactosidase A (α-GAL), glucosylceramide (GL1), globotriaosylceramide (GL3).

Drug Name	Mechanism of Action	Route of Administration	Physiological Effect
Agalsidase-β	Recombinant α-GAL	Intravenous infusion	Decreases accumulation of GL3
Agalsidase-α	Recombinant α-GAL	Intravenous infusion	Decreases accumulation of GL3
Migalastat	Binds reversibly to the active site of the amenable mutant of α-GAL	Oral	Promotes trafficking of α-GAL to lysosome, thus increasing enzyme activity
*Investigational drugs*			
Pegunigalsidase-α	Plant derived α-GAL	Intravenous infusion	Decreases accumulation of GL3
Moss α-GAL	Moss derived α-GAL	Intravenous infusion	Decreases accumulation of GL3
Lucerastat	Glucosylceramide synthase inhibitor	Oral	Reduces accumulation of glycosphingolipids, including GL1 and GL3
Venglustat	Glucosylceramide synthase inhibitor	Oral	Reduces accumulation of glycosphingolipids, including GL1 and GL3

**Table 3:** The European Fabry working group consensus document [46] recommendations for enzyme replacement therapy. enzyme replacement therapy (ERT), New York heart association (NYHA), maximal wall thickness (MWT), central nervous system (CNS), atrial fibrillation (AF).

Initiation of ERT	Discontinuation of ERT	Not a Candidate for ERT
Classical FD males: 16 years or older, even if they have no symptoms or clinical signs of organ involvement (Class IIB recommendation) Classical FD males and females: as soon as there are early signs of FD organ involvement (kidney, heart, and/or CNS signs) and not fully explained by other pathology (Class I recommendation). Non-classical FD males: as soon as there are early signs of FD organ involvement (kidney, heart, and/or CNS signs and not fully explained by other pathology (Class I recommendation). Non-classical FD females: early clinical signs consistent with FD (Class IIB recommendation) Cardiac-specific criteria: myocardial hypertrophy (MWT > 12 mm) without (or only minimal signs of) fibrosis (Class I) or signs of cardiac rhythm disturbances, including sinus bradycardia, AF, and repolarization disorders (Class I)	Noncompliance with >50 percent of treatments or patient request (Class 1). End-stage renal disease, without an option for renal transplantation, in combination with advanced heart failure (NYHA class IV)–Class IIA End-stage FD or other comorbidities with a life expectancy of <1 year-Class IIB The severe cognitive decline of any cause-Class IIB Lack of response for 1 year when the sole indication for ERT is neuropathic pain while receiving maximum supportive care. An exception is classical FD males who are at high risk of developing clinical signs of organ involvement within a short time period (Class II B).	Advanced cardiac disease with extensive fibrosis-Class I End-stage renal disease, without an option for renal transplantation, in combination with advanced heart failure (NYHA class IV)-Class IIA End-stage FD or other comorbidities with a life expectancy of <1 year-Class IIB The severe cognitive decline of any cause-Class IIB

## Data Availability

Data is contained in the article.

## Abbreviations

Fabry disease (FD), α-galactosidase A (α-GAL), globotriaosylceramide (GL3), globotriaosylsphingosine (lyso-GL3), cardiac magnetic resonance imaging (CMR), transthoracic echocardiography (TTE), left ventricular hypertrophy (LVH), left ventricular mass index (LVMi), enzyme replacement therapy (ERT), estimated glomerular filtration rate(eGFR), late gadolinium enhancement (LGE), extracellular volume (ECV), clustered regularly interspaced palindromic repeats (CRISPR).
